# Systemic Lectin-Glycan Interaction of Pathogenic Enteric Bacteria in the Gastrointestinal Tract

**DOI:** 10.3390/ijms23031451

**Published:** 2022-01-27

**Authors:** Seung-Hak Cho, Jun-young Park, Cheorl-Ho Kim

**Affiliations:** 1 Division of Zoonotic and Vector Borne Disease Research, Center for Infectious Disease Research, Korea National Institute of Health, Cheongju 28160, Korea; skcho38@korea.kr (S.-H.C.); wnsdud2057@korea.kr (J.-y.P.); 2 Glycobiology Unit, Department of Biological Science, Sung Kyunkwan University, Suwon 16419, Korea

**Keywords:** lectin, glycan, intestinal pathogenic bacteria, gastrointestinal tract

## Abstract

Microorganisms, such as bacteria, viruses, and fungi, and host cells, such as plants and animals, have carbohydrate chains and lectins that reciprocally recognize one another. In hosts, the defense system is activated upon non-self-pattern recognition of microbial pathogen-associated molecular patterns. These are present in Gram-negative and Gram-positive bacteria and fungi. Glycan-based PAMPs are bound to a class of lectins that are widely distributed among eukaryotes. The first step of bacterial infection in humans is the adhesion of the pathogen’s lectin-like proteins to the outer membrane surfaces of host cells, which are composed of glycans. Microbes and hosts binding to each other specifically is of critical importance. The adhesion factors used between pathogens and hosts remain unknown; therefore, research is needed to identify these factors to prevent intestinal infection or treat it in its early stages. This review aims to present a vision for the prevention and treatment of infectious diseases by identifying the role of the host glycans in the immune response against pathogenic intestinal bacteria through studies on the lectin-glycan interaction.

## 1. Introduction

Carbohydrates are not only the most important energy source in the living body, in the form of glucose and glycogen, but are also a biomaterial that plays a pivotal role in many biological processes, such as cell fusion, growth, adhesion, migration, death, and immune responses. These processes are largely performed through interactions between specific lectins, plasma membranes, and cell walls [1,2,3,4]. The carbohydrate components are supplied via the autotrophic biosynthesis of the photosynthetic pathway in plants and the consequent heterotrophic modification of glucose residues to various forms of monosaccharides. Comprehensive biosynthesis and modification of carbohydrates have recently been described in several studies [5].

Glycosylation is a post-translational modification that is involved in intracellular life processes, such as protein activity, intracellular signal transduction, and cell-to-cell interactions through attachment and detachment of glycans to proteins, lipids, and cell surfaces. Aberrant glycosylation reactions are known to be key mechanisms behind many diseases in humans [5]. In addition, glycosylated macromolecules are components of the cell wall and membrane, and they mediate cell-cell interactions. Research on the various forms of sugar generated by glycosylation is known as glycomics [6]. These sugars are present as conjugates with other substances, such as glycoproteins and glycolipids. Studies have shown that sugar chains are involved in most diseases; death may occur if part of the sugar chains are missing or abnormal, and sugar chains are related to bacterial infection and immunosuppression, the pathogenesis and progression of cancer, and metastasis [6,7]. It has recently been revealed that sugar chains are involved in various bacterial, fungal, and viral diseases. Studies have been conducted on lectins that bind to glycoproteins, glycolipids, oligosaccharyl glycans, and glycoconjugates [7].

Innate and adaptive immunity protect the host against infections. Pathogen-associated molecular patterns (PAMPs) are quickly recognized by the innate immune system. The known PAMPs are pili, lipopolysaccharides, flagella, peptidoglycans, and glucans. These are evolutionarily conserved in pathogenic bacteria, viruses, fungi, and protists [6]. PAMPs interact at the molecular level with lectin proteins, such as C-type lectins, galectins, and Siglecs, which are distributed among plants, invertebrates, and vertebrates [1,8].

Lectins are proteins that can bind to sugar chains in microorganisms, plants, and animals. Lectins are involved in various life phenomena such as protein quality control, host-pathogen interactions, cell-cell communication, inflammation, immune response, cancer progression, and development, through specific binding with sugar chains in living organisms. In fungi, lectins have specific affinities for glycoproteins and simple sugars. Recent studies have revealed that microfungal lectins play an important role in triggering the host immune response during pathogen-host interactions [9,10,11,12,13,14]. The encoded glycans and lectins are used by the host genome to inhibit the replication and spread of viral pathogens. A recent study reported that glycans and lectins control viral spread and immune system activation through virus-host interactions. Microbial infections are inhibited by inhibiting microbial adhesion. The mechanism of microbial infection involves the lectin and sugar chain-mediated biochemical and physiological metabolism of organisms, as well as the sugar chain and sugar chain-binding lectin. Glycomics research, including the discovery of alternative substances for antibiotics, separation and analysis of sugar chains, glycan microarray technology, and lectin-based cell imaging technology, are expected to be of increasing interest [15,16,17].

Research into sugar chains is much more complex and diverse than genome and protein research. Therefore, there have only been a few studies on glycan identification and prevention of diseases [18]. Research on the identification of adhesion factors involved in pathogen-host cell interactions in epithelial cells has been inadequate so far. In addition, results obtained from glycan-based pathogenesis and infectious disease manifestations are limited. Most studies on the pathogenesis of infectious bacteria have focused on the regulation of the expression of pathogenic factors after the onset of symptoms. To initiate studies on glycan-based pathogenicity, we previously reported a methodologic and multi-omics analytical construction using a lectin-glycan interaction (LGI) concept, applied to enterohemorrhagic *Escherichia coli* [19]. To expand on systemic LGI in infectious diseases, we discuss the methods required for the prevention and treatment of infectious diseases, focusing on the LGIs in the immune response of host glycans against pathogenic bacteria in the gut.

## 2. Lectin-Glycan Interactions

### 2.1. Ecosystems in the Human Gastrointestinal Tract (GIT) in Relation to LGI

**Figure 1:**
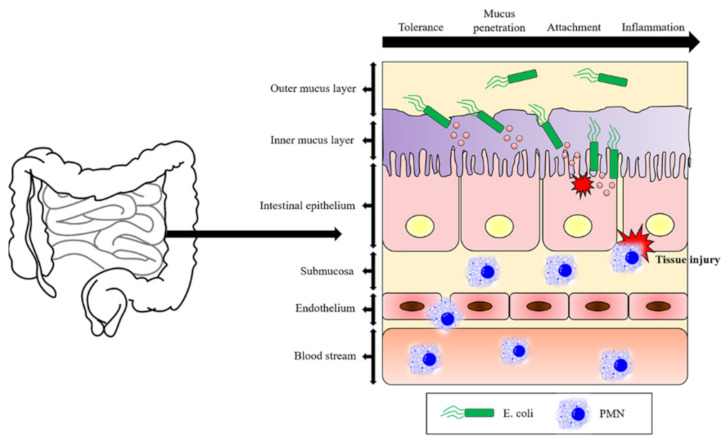
Mechanism of the pathogenesis of bacterial infections. Pathogenic bacteria reach the intestinal epithelial cells. They adhere to the layered glycan that exists in large quantities in intestinal epithelial cells via lectin interaction.

In the human intestine, there is a thick mucus layer (~700 µm) on entero-epithelial cells. In the mucus layer, there are substances that can protect against invading pathogens. The molecules found in the extracellular area include mucin, antibodies, defensins, protegrins, collectins, cathlecidins, lysozyme, and histatins. Normally, the intestinal microbiome lives peacefully in the mucus layer; however, when external pathogens destroy the mucus layer, they move toward the intestinal entero-epithelial cells. When pathogenic bacteria reach intestinal epithelial cells, they adhere to the abundant layers in the intestinal epithelial cells via lectin interactions. After pathogenic bacteria attach to such layers, the immune system works to fight infection (Figure 1) [20,21,22,23].

**Figure 2:**
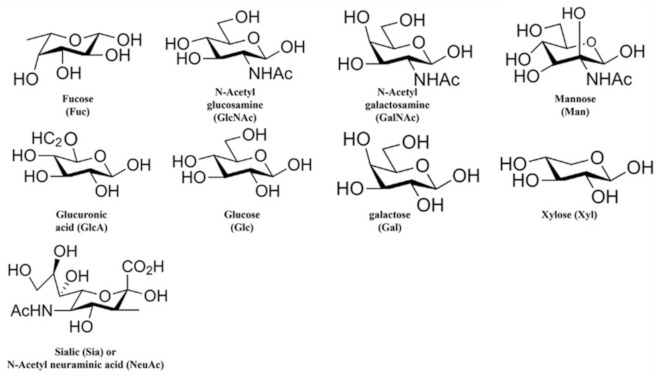
Structures of common monosaccharides. Structures of nine commonly found monosaccharides. Human glycan is composed of these nine key monosaccharides.

Glycans have a structure in which two to six monosaccharides are connected in a straight line; these moieties possess residual substances added via hydroxylation, phosphorylation, sulfation, methylation, and acetylation [24]. In O-glycans, the sugar linkage begins with N-acetylgalactosamine (GalNAc), in which serine and threonine amino acids are repeatedly linked, while in N-glycans, oligosaccharides with two N-acetylglucosamine (GlcNAc), nine mannose (Man), and three glucose (Glc) molecules are linked to asparagine (Asn) residues (Figure 2). After endogenous processing of glycans, nine carbon monosaccharides and sialic acid (Sia) are terminally attached to galactose (Gal) or GalNac residues.

Sia is a monosaccharide composed of nine carbons and is expressed by pathogenic bacteria and vertebrates. Since it is present at the end of the glycan structure, it is frequently present in host-pathogen interactions. In intestinal epithelial cells, a large amount of ganglioside is formed by the glycosphingolipid complex of one or several sialic acids in this basic sugar structure [25,26,27,28,29]. Recent studies have demonstrated that sugar chains bind proteins and lipids in vivo to regulate their functions [30]. For example, in intestinal enterohemorrhagic *Escherichia coli* (EHEC) infection, the carbohydrate structure and the protein surface are altered, resulting in a change in the binding of proteins to glycolipid sugar chains or interactions between proteins, leading to abnormal protein function. Previous studies have focused on Sia’s function as a biomarker for diagnosing infectious diseases by analyzing the structural changes in sugar chains that appear during infection [31]. Recent research has shown that glycolipid sugar chains directly induce and exacerbate infectious diseases [32].

### 2.2. Immune Responses in the GIT of Hosts in Connection with LGI

Recent studies have reported that the sugar chains in the glycolipids and glycoproteins of the host cells (glycocalyx) directly act on infectious pathogens in the early stages of infection [33,34].

The LGI network contains a variety of lectins and glycans (Table 1 and Table 2). Siglec is a kind of lectin that interacts with bacterial glycans. Cells can connect to one another through glycan-Siglec interactions, allowing signaling or, in the case of sialoadhesin, pathogen absorption. This protein has been found to be important in the phagocytosis of bacteria with heavily sialylated glycan structures, such as those in the *Neisseria meningitidis* lipopolysaccharides [35]. Macrophages that bind to these structures can phagocytose these bacteria, ridding the system of pathogens [36,37,38]. Siglec-7 is also involved in pathogen binding, such as of *Campylobacter jejuni*. This binding is sialic acid-dependent, and it brings bacteria into contact with NK cells and monocytes that express Siglec-7 [39]. These foreign pathogens can then be killed by the NK cells.

Siglec is a Sia-recognizing immunoglobulin-like receptor expressed in the major leukocytes of mammals. It recognizes Sia epitopes of glycoconjugates that are widely present on the surface of pathogens and regulates immune function and inflammatory activity against pathogens. Siglecs can be divided into 2 groups in terms of their sequence similarity and evolution: (1) Siglecs such as sialoadhesin, Siglec-2, -4, and -15 are common in mammals, as are (2) CD33-related Siglecs (CD33rSiglecs) [40]. An immunoreceptor tyrosine-based inhibitory motif (ITIM) is found in the cytoplasmic area of Siglecs at both the center and end of the membrane [41]. Cell growth, cytokine production, cell activation, and induction of apoptosis have all been shown to be negatively regulated by Siglec-containing ITIMs. Sia is detected by self-associated molecular patterns by CD33rSiglecs, thereby maintaining a basal inactive state for innate immune cells, resulting in a counter-regulated inflammatory response to the immune response It has been reported that pretreatment of the Siglec gene transfectants with sialidase enhances trans-ligand-binding activity [42]. CD33-rSiglecs are inhibitory receptors that lower the bactericidal activity of leukocytes against pathogenic bacteria, such as group B *Streptococcus* (GBS).

However, the signaling domain of sialoadhesin is lacking, and it contains only the 17 extended Ig-like extracellular domains, which prevents the potential cis-ligand masking of the Sia-binding part from rising out of the surface glycocalyx [43,44]. Sialoadhesin is a receptor for phagocytosis by macrophages and promotes effective phagocytosis of GBS and other sialylated pathogens. Sialoadhesin induces phagocytosis or endocytosis by initial adhesion to sialylated pathogens, thereby enabling the effective elimination of pathogens in response to infection.

Many pathogenic bacteria use Sia as an anti-recognition substance to avoid the host’s immune response. Thus, future studies should aim to determine whether sialylated pathogenic bacteria utilize the host’s receptor system to modulate the immune response. The function of Siglec is a promising research area; studies on Siglec and its binding specificities could be key to preventing or treating disorders, such as chronic obstructive pulmonary disease or premature birth.

### 2.3. Outline and Present Status of LGI Research

Since lectins recognize and bind to sugar chains, they can be utilized in the development of antibody technologies. Lectins may also be used for recognition, separation, and purification of biomolecules related to various sugar complexes and for ELISAs, mutant cell selection, blood typing, histochemical staining, glycosyltransferase or glycosidase assays, cell sorting, and glycoconjugate purification. Using lectin specificity, early measurement and diagnosis of changes in glycoproteins, glycolipids, and oligosaccharides present on the cell surface during the onset of specific diseases have been made, and various lectins, sugar chains, viruses, and microorganisms binding to cell surfaces have been characterized [45]. Immune LGIs use aggregation and agglutination to target the sugar chains on the surface by host-expressed lectins [46]. An alternative glycan-based strategy to treat and prevent infectious and non-infectious diseases is to use various fluorescent probes for the synthesis and selective binding of lectin to sugar chains. Sugar chains present on certain cell surfaces, viruses, and microorganisms are easily recognized by these lectin probes. In certain cancer cells, specific intracellular proteins can be targeted and measured at an early stage [47]. Fluorescent lectin probes have been used extensively to determine the location of cells, interactions between cells, and the invasion of viruses and microorganisms into cells through optical microscopy and have been used to measure images of living organisms through sugar structures [48]. The fluorescent lectins enable the simple detection of tumor cells and extracellular vesicles without the need for purification of the surface glycan moieties, as lectins recognize the glycan.

Many bacterial lectins mediate adhesion to host cells as adhesins, defining bacterial cell and tissue affinity [49,50,51,52]. Recently, studies on the LGI of several types of bacteria, such as *E. coli*, *Helicobacter pylori*, group A *Streptococcus*, and *Neisseria gonorrhoeae,* have been published [53,54]. However, the glycans that bind to the bacterial lectins have not yet been determined, and systemic lectin discovery and application studies have not yet been conducted. Although research on host-pathogen interactions is actively being conducted, studies on LGI in pathogenic bacteria are in an early stage.

### 2.4. Strategies to Search for Lectins of Pathogenic Intestinal Bacteria

**Figure 3:**
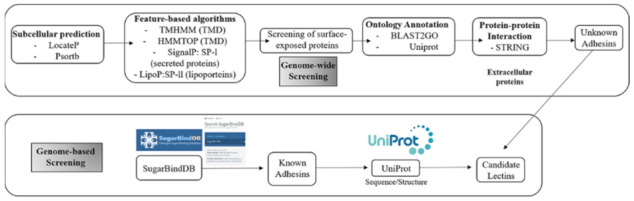
Workflow of gene-based and genome-wide screening methods for finding lectins of pathogenic bacteria. Gene-based screening is a method for finding known adhesion factors using the SugarBindDB program, and genome-wide screening is used to find unknown adhesion factors using bioinformatics.

Studies investigating the lectins of pathogenic intestinal bacteria can be divided into those that follow non-experimental and experimental methods. Non-experimental methods for finding lectins from pathogenic bacteria include searching for known adhesion factors using the SugarBindDB program (gene-based screening) and investigating unknown adhesion factors using bioinformatics (genome-wide screening) (Figure 3).

Figure 3 (top) shows how one might find new adhesin candidates. First, for a species whose full-length genome sequence is known, or at least some gene sequence is available, subcellular localization is predicted from sequence information and annotation information to search for surface proteins, and the type of protein (secreted protein, lipoprotein, etc.) and domain structure (transmembrane domain [TMD]) can be confirmed using a feature-based algorithm. The function of the selected surface protein can be predicted through ontology annotation, and the proteins that interact with glycoprotein can be identified by searching the STRING database for the interactions of the protein whose function was predicted using databases, such as BLAST2GO and UniProt. PSORTb software is used to predict outer membrane adhesins from annotated genes in *E. coli* strains. A target outer membrane protein located in the outer membrane is predicted based on intracellular localization analysis using *E. coli* genetic information, and a candidate outer membrane protein, which is a lipid protein, is predicted as the *E. coli* outer membrane protein by using a specific formula [55,56]. Figure 3 (bottom) shows a gene-based screening method that is used to search for known adhesins in SugarBindDB. Among the adhesins of the target species, known adhesins are searched in SugarBindDB, and sequence information is retrieved from the UniProt database and used as a candidate lectin [57,58,59,60].

Another method is to use a simulation program based on computational chemistry. The docking simulation, which predicts the structures of sugar chains that can bind to a lectin ligand, is a crucial technique in computational chemistry [61]. These protein-ligand or sugar chain-lectin ligand docking programs are used to understand the functions of the ligands by predicting their binding structure. These programs are also used to discover and optimize the binding site and candidate materials [62,63,64]. It is essential to predict the binding pose with high accuracy. The current algorithm “cuts” the target ligand and predicts the location of the fragment by matching it with the fragment clusters in the reference library. Possible hydrogen bonds, hydrophobic interactions, and non-bonded contact characteristics are utilized as default settings based on the docking simulation findings.

**Figure 4:**
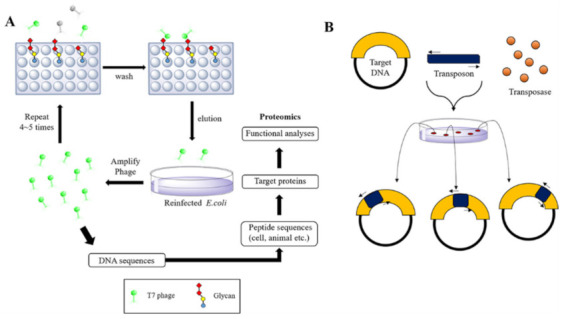
T7 phage display and transposon mutagenesis methods for bacterial lectin selection. (**A**) Phage display is a method used to identify binding peptides or proteins which are expressed in the phage capsid. (**B**) Transposon mutagenesis is the process of disrupting genes by randomly inserting a fragment of DNA, termed a transposon, into the genome.

Phage display is an experimental method used to identify binding peptides or proteins. The test protein is expressed in the T7 phage capsid; the T7 phage is tough and stable, making it easy to use [65]. As it has a cloning vector that contains a multi-cloning site, it is compatible with other general vectors, so it is easy to clone. In the T7 phage display method to find bacterial lectin, the phage is used to infect bacteria, attached to a bead covered with glycan, and is thus displayed. The DNA of the adhered phage is sequenced, and its protein is revealed [66,67,68] (Figure 4A).

Transposon mutagenesis is another experimental approach for disrupting genes by randomly inserting a fragment of DNA, called a transposon, into the genome (Figure 4B). A variant TNP gene encodes the plasmid-borne transposase (Tnp), which inserts the transposon into the recipient strain’s genome with very minimal insertional bias [69,70,71]. As a result of this strategy, vast mutant libraries containing transposons inserted into unique genomic locations in a recipient strain of *E. coli* or other bacteria have been created [72]. Large libraries with hundreds of thousands of distinct clones may be generated using bacterial conjugation rather than other methods, like electroporation or chemical transformation. Using these libraries, this approach may be used to find lectin-binding domains.

Carbohydrates are synthesized by specific enzymes without a template; unlike DNA or proteins, mass production is difficult, and it is also difficult to isolate with high purity. For this reason, there is an urgent need for technology that can perform analyses using small quantities of carbohydrates. Various methods, such as mass spectrometry, high-performance liquid chromatography, and frontal affinity chromatography (FAC) are used to analyze cell surface glycans [73]. Although such analysis techniques can provide more accurate structural and quantitative information on sugars, they require large quantities of samples for analysis, which can make it a complicated and time-consuming process. Therefore, microarray-based technologies that can quickly analyze glycoproteins or sugars on the cell surface can fill the shortcomings of the above-mentioned techniques [74].

**Figure 5:**
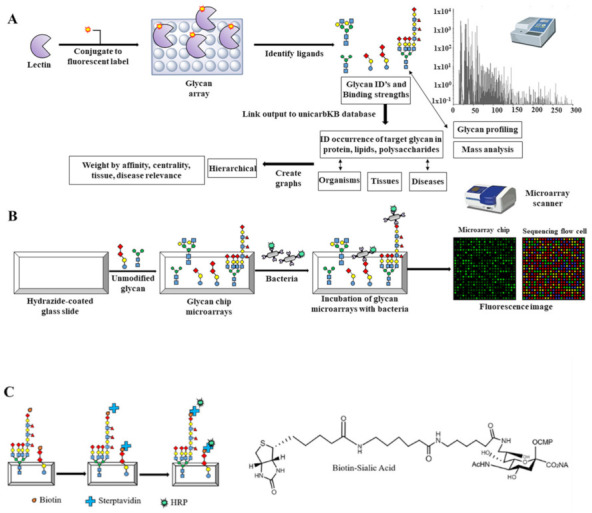
Glycan microarray methods for finding the specificity of the pathogen’s lectin-carbohydrate interactions. (**A**,**B**) By binding to the bacterial surface lectin, a glycan microarray-based technique for screening functional glycans was developed. Microscopy and/or a microarray scanner measure the intensity of each spot’s fluorescence. (**C**) The microarray is treated with streptavidin-labeled HRP and biotinylated glycan (sialic acid). The many biotins of the biomolecular complex bind to a Cy3- or Cy5-labeled streptavidin, resulting in signal amplification.

Microarray technology can quickly and simultaneously analyze the interactions of thousands of samples and requires only a small amount of each sample. To determine the potential host glycoprotein attached to the pathogen adhesin, it is possible to qualitatively or quantitatively analyze the specificity of the lectin of the pathogen for the host glycan by glycan array screening (Figure 5A,B) [75,76]. Biotinylated glycans are a new family of carbohydrate or glycoconjugate probes that make use of avidin’s and streptavidin’s strong affinity for biotin. The multiple biotins of the biomolecular complex are permitted to bind to Cy3- or Cy5-labeled streptavidin, resulting in signal amplification (Figure 5C) [77,78,79]. Biotinylated sugars are stable and reliable for research on protein-carbohydrate interactions, ranging from simple O-linked monosaccharides to a more complicated N-link biantennary system.

Identification of lectin-glycan interactions of human glycolipid-binding bacteria-derived proteins is an important research topic for the development of adhesion factor inhibitors and preventive treatments. For this research, it is necessary to identify the structure of the lectin of the pathogen and accurately determine the point of attachment to the receptor of the host. For example, information on the attachment site to host glycoprotein in the structure of FimH is well known [80].

**Figure 6:**
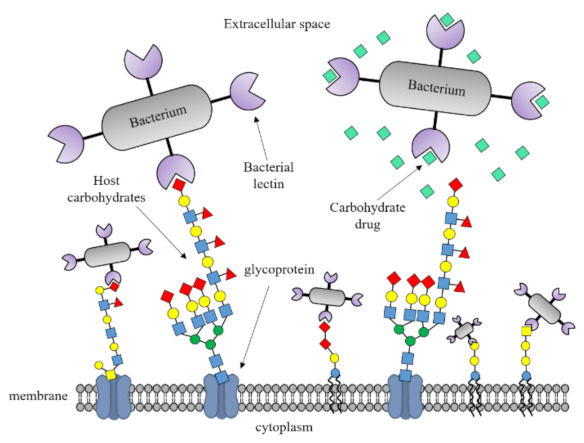
Schematic representation of lectin-glycan interactions in bacterial pathogenesis. Infectious diseases can be prevented and treated by identifying the role of the host glycans in the immune response against pathogenic intestinal bacteria through studies on the lectin-glycan interaction.

As shown in Figure 6, if the protein sequence and structure of the initial adhesion factor (lectin) of the enteric pathogen are determined, and an inhibitor that attaches to the lectin is developed, intestinal infection can be prevented. This could function as an antibiotic replacement treatment that can be used for treatment in the early stages of infection.

## 3. Conclusions

### Future Perspectives of LGI Research

There have been several reports on the interactions between pathogen lectins and host glycans during the initial stages of infection. Protein sequences and structures of the pathogen lectins can be the basic data needed to develop a vaccine or antibiotic alternative that can be applied to clinical practice. Such a product will have much public health and economic impact by reducing treatment costs for intestinal infections. Furthermore, we believe that fundamental creative discoveries and innovative research strategies on lectins, glycans, and their translations, as a basis for ultimately protecting and improving health, are a promising direction for future research.

## Figures and Tables

**Table 1:** LGI adhesion factor selection through gene-based screening.

Lectin	Bacteria	Glycan
Shiga toxin	EHEC	Globotriaosylceramide (Gb3)
FedF	EHEC	H type 1, A6 type 1, H5 type 1, A6 type 1, Antigen H
*E. coli* K99 fimbriae	ETEC	NeuGc-GM3 (GM3gc)
CS3	ETEC	GalNac (β1-4) Gal
20 K fimbriae (CS31A surface adhesin)	ETEC	N-acetylglucosamine (GlcNAc)
S-fimbrial adhesin	ETEC	Siaα2-3Gal
Fimbrial adhesins	ETEC	Mannose (Man)
Bacterial adhesins	ETEC	Galabiose (Ga2), Siaα2-3Gal
Bacterial adhesin F17-G	ETEC	N-acetylglucosamine (GlcNAc)
Fimbriae Recognizing Sialyl Galactosides	ETEC	Siaα2-3Gal
FimH	ETEC	Mannose (Man)
LT	ETEC	NeuAc-GM1, NeuAc-GD1b, Asialo-GM1a
F4 fimbriae	ETEC	Lactosylceramide (LacCer), Globotriaosylceramide (Gb3), Hexaosylceramide, sulfatide, Asialo-GM2, Asialo-GM1, Galablosylceramide

**Table 2:** LGI adhesins used by bacteria to colonize the mucus layer.

Lectin	Bacteria	Glycan
Peb3	*Campylobacter jejuni*	phosphoenolpyruvate, 3-phosphoglycerate
BC2L-C Nter	*Burkholderia cenocepacia*	H type 1 and Leb
BC2L-C Cter	*Burkholderia cenocepacia*	α-Man and a-mannoheptulose
Hif fimbriae	*Haemophilus influenzae*	α-Neup5Ac-(2-6)-β-Galp-(1-4)-GlcpNAc
CupB6	*Pseudomonas aeruginosa*	Leb
LecA/PA-IL	*Pseudomonas aeruginosa*	α-Galp-(1-3)-β-Galp-(1-4)
LecB/PA-IIL	*Pseudomonas aeruginosa*	α-Fucp-(1-3/4)-β-GlcpNAc
BabA	*Helicobacter pylori*	Leb, A/H type 1, Globo A/H
SabA	*Helicobacter pylori*	α-Neup5Ac-(2-3)-β-Galp-(1-4)-b-GlcpNAc
FaeG	*Escherichia coli*	α-GalpNAc-(1-3)-β-GalpNAc-(1-3)-β-Galp-(1-4)- β-Glcp-(1-O)-Cer
FedF	*Escherichia coli*	ABH type 1 (Figure 2), sulphated H type 2
PapG	*Escherichia coli*	β-GalpNAc-(1-3)-a-Galp-(1-4)- β-Glcp-(1-O)-Cer

## Data Availability

Not applicable.
